# Community and District Empowerment for Scale-up (CODES): a complex district-level management intervention to improve child survival in Uganda: study protocol for a randomized controlled trial

**DOI:** 10.1186/s13063-016-1241-4

**Published:** 2016-03-11

**Authors:** Peter Waiswa, Thomas O’Connell, Danstan Bagenda, Pricila Mullachery, Flavia Mpanga, Dorcus Kiwanuka Henriksson, Anne Ruhweza Katahoire, Eric Ssegujja, Anthony K. Mbonye, Stefan Swartling Peterson

**Affiliations:** School of Public Health, Makerere University College of Health Sciences, Kampala, Uganda; Karolinska Institutet, Solna, Sweden; Economics and Finance, UNICEF NewYork, Three UN Plaza, New York, NY 10017 USA; College of Public Health, University of Nebraska Medical Center, Omaha, NE USA; Harvard T.H. Chan School of Public Health, Harvard University, Boston, MA USA; UNICEF NewYork, Three UN Plaza, New York, NY 10017 USA; Health Section, UNICEF Uganda Country Office, Box 7074, Kampala, Uganda; Uppsala University, Uppsala, Sweden; Child Health and Development Centre, Makerere University, Kampala, Uganda; Director Health Services, Ministry of Health, Box 7272, Kampala, Uganda

**Keywords:** Child survival, Management tools, Bottleneck analysis, Evidence-based, District strengthening, Community monitoring, Health systems strengthening, LQAS, Uganda

## Abstract

**Background:**

Innovative and sustainable strategies to strengthen districts and other sub-national health systems and management are urgently required to reduce child mortality. Although highly effective evidence-based and affordable child survival interventions are well-known, at the district level, lack of data, motivation, analytic and planning capacity often impedes prioritization and management weaknesses impede implementation. The Community and District Empowerment for Scale-up (CODES) project is a complex management intervention designed to test whether districts when empowered with data and management tools can prioritize and implement evidence-based child survival interventions equitably.

**Methods:**

The CODES strategy combines management, diagnostic, and evaluation tools to identify and analyze the causes of bottlenecks to implementation, build capacity of district management teams to implement context-specific solutions, and to foster community monitoring and social accountability to increase demand for services. CODES combines UNICEF tools designed to systematize priority setting, allocation of resources and problem solving with Community dialogues based on Citizen Report Cards and U-Reports used to engage and empower communities in monitoring health service provision and to demand for quality services. Implementation and all data collection will be by the districts teams or local Community-based Organizations who will be supported by two local implementing partners. The study will be evaluated as a cluster randomized trial with eight intervention and eight comparison districts over a period of 3 years. Evaluation will focus on differences in uptake of child survival interventions and will follow an intention-to-treat analysis. We will also document and analyze experiences in implementation including changes in management practices.

**Discussion:**

By increasing the District Health Management Teams’ capacity to prioritize and implement context-specific solutions, and empowering communities to become active partners in service delivery, coverage of child survival interventions will increase. Lessons learned on strengthening district-level managerial capacities and mechanisms for community monitoring may have implications, not only in Uganda but also in other similar settings, especially with regard to accelerating effective coverage of key child survival interventions using locally available resources.

**Trial registration number:**

ISRCTN15705788, Date of registration; 24 July 2015.

## Background

Despite substantial progress in the health of the under-5s at the end of the Millenium Development Goals (MDGs) in 2015, mortality in sub-Saharan Africa (SSA) remains high [1]. In order for countries to make further and rapid progress, there is urgent need to scale up evidence-based interventions. In many SSA countries, Uganda inclusive, district health systems remain the mainstay of implementation and scale up of evidence-based interventions. However, in many instances district health systems are weak and fail to use limited resources to equitably implement, with high coverage, quality evidence-based interventions.

Previous studies have demonstrated that the failure to succeed in scaling up child survival interventions is mainly due to several factors at the sub-national level, including: (1) lack of supportive policies, (2) failure to prioritize those interventions that are most likely to prevent deaths, (3) problems with the essential commodities for vaccination services and treatment of illnesses, (4) absence of community-based health promotion activities, and (5) bottlenecks to the timely provision of primary health care prevention and curative services for the main causes of mortality [2].

In addition, lack of disaggregated data on bottlenecks to service coverage and tools to assess performance prevents the identification of inequalities within the districts and the definition of priorities for action. The weak support for implementation of interventions at the district level is attributed to factors such as poor management capacity, including the lack of local abilities and local data to prioritize and contextualize interventions, insufficient emphasis on results, lack of identification of health system bottlenecks to effective coverage, lack of needs-based financing and resources allocation to carry out context-specific managerial solutions and lack of financial decision-making space to carry out context-specific managerial solutions, and failure to involve communities as active proponents in helping overcome obstacles to high coverage [3–5]. The problem is compounded by the populations’ being dissatisfied with the poor quality of public services, insufficient information provided by staff, and gaps in health worker behavior and coping mechanisms [6]. A key knowledge gap is how, amidst limited resources, districts can be empowered to prioritize, plan and implement evidence-based child survival interventions.

With the above in mind, and with respect to the main childhood killers – malaria, pneumonia, and diarrhea, we designed the Community and District Empowerment for Scale-up (CODES) intervention and plan to test its application at district level. The CODES intervention endeavors to prioritize and address the bottlenecks (management and implementation challenges) that hinder effective quality coverage using a strategy that is based on three pillars including:

1. The application of a novel approach to help identify priority pneumonia, diarrhea, and malaria interventions using local data, identify supply and demand bottlenecks in the coverage of interventions, and assess gaps between resource needs and availability
2. The combined provision of mentoring and peer-to-peer support in a quality improvement approach combined with other management tools and targeted funding to eliminate identified bottlenecks
3. The increased community involvement in on-going assessments of quality and access barriers, and the mobilization of communities to improve community practices and care-seeking behaviors

We hypothesized that districts receiving the CODES intervention will have significantly higher coverage of key protective, preventive, and curative indicators (Table 1) for pneumonia, diarrhea and malaria compared to comparison districts.

**Table 1:** Protect prevent and treat interventions considered for primary outcome indictors

	Protect by children establishing good health practices from birth	Prevent children becoming ill from malaria, pneumonia and diarrhea	Treat children who are ill with malaria, pneumonia and diarrhea with appropriate treatment
Acute respiratory infection	1. Exclusive breastfeeding for 6 months	1. Vaccines: measles, Hib. PCV	1. Improved care seeking and referral
	2. Adequate complementary feeding	2. Use of LLINs	2. Timely appropriate case management at health facility and community level
	3. Vitamin A supplementation	3. Hand washing with soap	3. Availability of key supplies: ACTs, RDTs, ORS, zinc, antibiotics
Diarrhea		4. Safe drinking water	4. Continued feeding (including breastfeeding)
		5. Improved sanitation	
		6. (Reduced household pollution)	
		7. HIV prevention	
Malaria/Fever		8. (Cotrimoxazole prophylaxis for HIV- infected exposed children as HIV prevention)	
		9. Intermittent presumptive treatment	

*ACT HiB Haemophilus influenzae* type b, *HIV* human immunodeficiency virus, *LLIN* long-lasting insecticidal nets, *ORS* oral rehydration solution, *PCV* pneumococcal conjugate vaccine, *RDTs* rapid diagnostic tests

## Methods

### Trial design

This is being implemented as a cluster randomized controlled trial with eight districts (clusters) as intervention and eight other districts as comparison districts. All the then 111 districts in Uganda were assessed for eligibility, but 81 of those did not meet the eligibility criteria of having a high burden of child mortality threshold as set by United Nations Children’s emergency Fund (UNICEF). Five eligible districts with a high mortality (clusters) that were involved in the proof-of-concept phase of the CODES intervention were also excluded for consideration in the randomization district sampling frame. At the analytical level all the individuals as defined by the respective target groups will be eligible for analysis.

### Trial setting and randomization

**Fig. 1:**
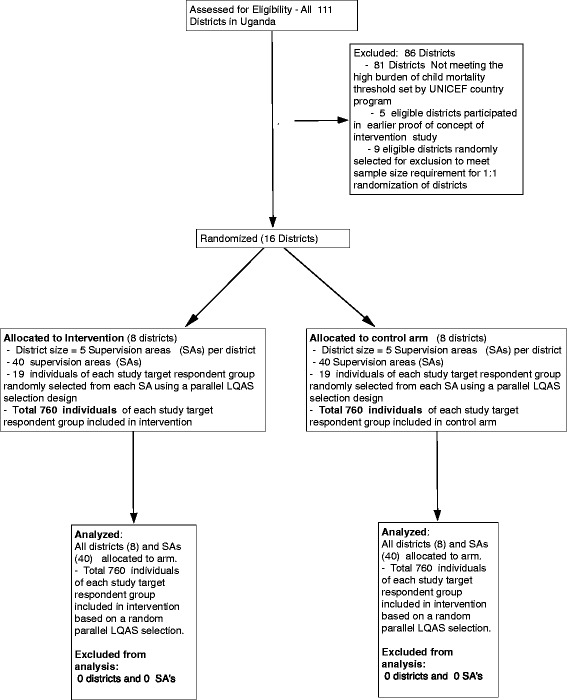
Trial profile for Community and District Empowerment for Scale-up (CODES)

The focus of CODES is at district level, which is increasingly the administrative unit at which implementation as well as decision-making is taking place [7]. The study is being undertaken in 16 districts in Uganda, which are randomly selected from a purposively selected sampling frame of 25 districts taken from the overall total of 111 districts in the country, because they have the highest burden of child mortality as determined by the country’s UNICEF office using data based on estimated absolute numbers of annual under-5s’ deaths. The districts in the sampling frame were stratified and/or matched on the basis of geographical region, recentness of establishment of district (defined by whether it was “created” as *a child district after 2010*), level of on-going district-level Lot Quality Assurance Survey (LQAS)-based quality improvement interventions and current district potential for reducing child mortality based on a dichotomized composite index. Eight pairs of districts were randomly selected from each of the stratified blocks that resulted from the matching obtained using the above criteria. Within each such pair and by coin toss, a district was randomly assigned to either obtain the CODES intervention or, as control to continue with their typical management and prioritization practice (Fig. 1).

The randomization was conducted independently by the monitoring and evaluation team based at Makerere University which does not participate in implementation and is independent of the implementing NGOs (Advocates Coalition for Development and Environment, ACODE, and Child Fund International, CFI). In addition, to improve the likelihood of balance in the randomization arms, nine eligible districts were randomly selected for exclusion to meet the sample size requirement of 1:1 randomization of districts.

### Sample size calculations

Based on the CODES pilot in five districts, and also according to data from the Uganda Demographic Health Surveys, the estimated baseline levels for the various key coverage and quality indicators for malaria, diarrhea and pneumonia ranged from 2 to 85 %. On the basis of these baseline estimates, sample size estimates were computed with a goal to achieve 80 % power of observing a greater than 25–30 % (regarded to be of public health significance) absolute difference in increase in various outcome indictors between intervention and control groups at the 5 % significance level (and assuming the typical level of intra-cluster correlation of 0.07 associated with most of the indicators). The computations revealed that a minimum of four districts (with each district providing a fixed LQAS-based evaluable sample of 95 individuals for each of the relevant target population) would have to be randomized per CODES study arm to address the primary objective assuming this cluster randomized design [8].

### The intervention

The CODES strategy is anchored on three pillars as earlier stated, these are:

1. Improved targeting of interventions and better allocation of resources to match disease burden
2. Use of evidence-based management tools and focal funding to overcome management bottlenecks in order to improve district health team performance
3. Community oversight and exerting accountability to ensure improvements in both coverage and quality of key interventions to reduce child deaths

In pillar 1 (improved targeting of interventions and better allocation of resources to match disease burden), we are using the LQAS methodology to collect data for use in understanding poorly performing indicators around evidence-based interventions. LQAS has the advantage that it is a programmatic methodology but its data can also be aggregated to look at broader progress in study areas [9]. The aggregated data from the baseline and endline LQAS surveys will also be used as the primary data for the analysis of the cluster randomized trial.

The LQAS data is collected from households, the village health team members (the community health workers in Uganda) and from health facilities. The project adapted the Tanahashi model [10], which, through input of locally generated data around coverage of key evidence-based interventions, is used to assess the system bottlenecks. The Tanahashi model recognizes the coverage of interventions, but goes further to emphasize “effective coverage”, which refers to coverage of sufficient quality to achieve the desired impact. The Tanahashi model focuses on six coverage indicators with three on the supply side (availability of essential commodities, relevant skilled human resources to offer services and the accessibility of these services through looking at the distance to the health facility from the service users) and three on the demand side (initial utilization, continuous utilization and effective utilization). After identifying the bottlenecks in coverage of interventions, a causal analysis will be done through brainstorming with the aim of reaching the root causes (the underlying phenomenon that led to the bottleneck) of the bottlenecks. It is hoped that identifying bottlenecks and their root causes should make evidence-based planning easier for managers and should address context specific district priorities.

In the second pillar, which is use of evidence-based management tools and focal funding to overcome management bottlenecks, the study will use the following management tools. The reason for this is that although LQAS surveys and the District Bottleneck Analysis (DBA) tool help in diagnosing problems, they do not identify potential contextualized solutions. For this reason, we adopted the UNICEF district management checklist tool to complement the DBA tool in order to assist district teams in conducting causal analysis to identify management problems to overcoming bottlenecks, and to provide evidence-based alternative solutions that can be adapted to the local context [11]. A variety of other methods and tools also exist to support District Health Management Teams (DHMTs) in the resolution of bottlenecks. These include peer-to-peer mentoring and regular assessment of performance with senior staff acting as peer reviewers, a method that has been linked to measurable improvements in coverage of health services, greater access, and more equitable health outcomes [10, 12–15]. One-time interventions to resolve bottlenecks are clearly not enough to resolve most problems. Furthermore, as problems are solved, others may be created or become more apparent, and it is thus important that the process of bottleneck resolution be a dynamic process. For this reason, quality management principles from the Model for Improvement (MFI) use so called “plan-do-study-act” cycles that allow tackling district management issues, at the district, sub-district, as well as at the facility level, to close performance gaps in small incremental, problem-solving steps [16, 17].

The third pillar is community oversight and exerting accountability to ensure improvements in both coverage and quality of key interventions to reduce child deaths. Here the demand side component of the CODES intervention will be implemented through use of facilitated community dialogues where communities will be empowered to demand for these services as well as exerting accountability. The CODES intervention will use community dialogues, Citizen Report Cards, and text message surveys to support overcoming demand side barriers as well as in improving health system accountability. Both qualitative and LQAS data will be used to develop a community score card, which in turn will be used by community members to demand for services and performance of providers. Linkages will be facilitated through mobile phone tools such as U-Report, Rapid SMS, community score cards and other community-based mechanisms, to feed into monitoring and evaluation of DHMT performance, and hold DHMTs accountable for improving access, coverage, and quality to ensure quality implementation of key interventions to reduce child deaths.

In the intervention districts, DHMTs will be encouraged to enhance accountability via an approach to solicit feedback from the community with assistance of local Community-based Organizations (CBOs). Community involvement in monitoring health providers and promoting community-based activities and social monitoring has been demonstrated to improve care-seeking and social accountability by service providers [18]. In addition, it has been shown that community processes can improve preventive and protective behaviors, as evidenced by the positive effects of participatory women’s groups doing action-learning cycles on perinatal health in Asia [19].

At the national level, the project performs media campaigns as well as engagement with policy-makers to devise solutions for national-level bottlenecks which cannot be addressed at the community level.

The CODES intervention will be introduced to each intervention district via its DHMTs through sensitization meetings, hands-on execution and mentorship with support from two NGO implementing partners – Child Fund International (CFI)/Liverpool School of Tropical Medicine (LSTM)) on the supply side and using Advocates Coalition for Development and Environment (ACODE) on the demand side.

Key activities to be implemented in all intervention districts include a disaggregated situational analysis in order to identify areas in the district with poor coverage on key indicators, underserved populations and bottlenecks to the delivery of health services. Comparison districts will receive the current government standard of care, that is, will have no systematic processes around the three CODES pillars. In other words these districts will not be facilitated to collect comprehensive LQAS and community data, to conduct Bottleneck Analysis (BNA) to identify priorities and align these to available resources in a systematic order, and may not have NGOs to facilitate Continuous Quality Improvement (CQI)implementation and community dialogues.

### Primary and secondary outcome measures

#### Primary outcomes

The primary outcome measures for comparison in this trial will be the coverage of key protective, preventive and curative quality coverage indicators for pneumonia, diarrhea, and malaria (Table 1). These quality and coverage indicators have been found to be the most effective interventions for pneumonia, diarrhea, and malaria across the protective, preventive and curative spectrum, and at community/family and health-facility/health care worker-level deaths.

#### Secondary outcomes

Other outcome measures that will be evaluated include the prevalence of pneumonia, diarrhea, and malaria amongst the target under-5 year age group. These might be suggestive of the status of protective and preventive interventions. Mortality impact will be a secondary indicator and will be modeled using the Lives Saved Tool (LiST) since reliable child mortality data is lacking at the district level and we are unable to measure it in this project [20]. We will also document the immediate management outcomes in the intervention arm such as the presence of annual reports that reflect priority bottlenecks for pneumonia, diarrhea, and malaria as determined by the bottleneck/causal analysis intervention tools. This will reflect the level of adherence to the intervention and to get a sense of how well the subgroup that carried out the intervention as intended was successful on the outcome indicators.

#### Data collection and analysis plan

Primary data collection will use parallel community (household) LQAS questionnaire-based surveys conducted in both intervention and control districts at baseline, midterm (2 years later), and at endline. In order to be pragmatic and to suit the Uganda Child Survival Package, in the intervention districts these LQAS surveys will also be the primary tool for collecting information to be used in the BNA. Each of the 16 districts included in the cluster randomized trial will be divided into five strata (supervision areas) and, based on a sample list of villages from the Uganda Bureau of Statistics (UBoS) for each strata, 19 villages will be randomly selected based on number of households per village using Probability Proportional to Size (PPS) methodology. A total of 760 individuals of each study target respondent group will be included in both the intervention and control arms.

In each village, a random reference household will be selected using a table of random numbers applied to a village household list or village sketch map, and the next nearest door to this will determine the first and subsequent household from which one randomly selected respondent from each of the target populations will be sought for interview with a maximum of one respondent from each household regardless of category.

The household LQAS surveys will focus on seven target population groups including mothers of children under 6 months, mothers of children 6–11 months, mothers of children 12–23 months, mothers of children under 5 years, mothers of children under 5 years with diarrhea in the last 2 weeks, mothers of children under 5 years with acute respiratory tract infections in the last 2 weeks and mothers of children under 5 years with fever in the last 2 weeks, resulting in 19 individuals from each target population group from each village (Fig. 1).

Information indicating adherence to CODES intervention will be based on documentation reports from the support implementing partners (CFI/LSTM and ACODE). To evaluate community participation and changes in demand side perceptions and behavior we will collect data by short messaging service (SMS) survey polls, LQAS surveys, and focus group discussions (FGDs).

Primary analysis for the cluster randomized trial will use an intention-to-treat approach to districts as either allocated to intervention or control group. We will conduct a difference of differences analysis of proportions of the key quality coverage indicators looking at differences between baseline and endline survey estimates for the intervention and for the control district areas. The analytical techniques used will appropriately take into consideration the clustering design effects due to the cluster randomized design, as well as any weighting factors, using techniques that will include but not be limited to generalized estimating equations (GEE) with analysis of covariance (ANCOVA).

Secondary analysis will look at a subgroup of districts that are deemed to have “performed well” on the basis of immediate outcomes (such as the annual report) to see if there is any indication of “dose-response.” Similarly, subgroups of vulnerable populations will be evaluated for coverage in an equity analysis. Based on the coverage achieved in the intervention and comparison districts, we will model the estimated lives saved using the LiST. Process indicators and contextual factors will also be documented to aid in the interpretation of the findings. To evaluate changes in management behavior among the DHMT, data collection will include participant observation, in-depth interviews with stakeholders, and analysis of documents such as district health plans and implementation monitoring reports. Qualitative data will be analyzed using content analysis.

Ethical clearance for the project was given by Uganda National Council for Science and Technology (Ref:SS2548). All districts will implement the Uganda Child Survival Strategy as per the Health Sector Strategic Plan. Informed consent will be obtained from each study participant after explaining the objectives of the study and procedure. Confidentiality of interviewees will be maintained. A study Steering Committee chaired by the Ministry of Health and involving key partners and the study teams has been set up to facilitate policy linkage. The Steering Committee meets quarterly.

## Discussion

Previous studies have highlighted the poor institutional capacity in decentralized health systems of developing countries [7, 21–23]. Decentralization in Uganda had the objective of bringing political power closer to local communities to respond to local needs, build local capacity, and improve accountability However, there are major constraints in the implementation of health interventions by district health management teams. CODES aims to increase coverage of effective interventions by increasing DHMT capacity to prioritize and implement context-specific solutions, and by empowering communities to become active partners in diagnosis and monitoring barriers to access and quality service provision, as well as in the equity of utilization.

If the CODES intervention is successful, an increase in effective coverage of child survival interventions is expected, and this should be associated with reductions in child mortality. We designed the main CODES trial after an initial pilot in five districts. This pilot phase enabled us to learn how to adopt, adapt, and harmonize the project intervention components which we are now testing using a controlled design.

However, we are aware that our design has limitations associated with the measurements and data collection techniques. The use of multiple data sources, i.e., government administrative databases as a source for supply side data and surveys as a source for demand side data, might produce different patterns of utilization and availability of services. For instance, a household survey will capture people that received service regardless of the type of provider (public or private), while administrative databases will provide only public sector data. Another limitation is the use of LQAS methodology to collect data. This data collection technique does not produce point estimates of coverage. It typically can be used only to assess the coverage in relation to a pre-established cut-off in each “lot.” However, at district and overall randomization arm level it is possible to calculate a coverage level by weighting the population size of each sub-district and aggregating these data together. In addition, we choose to use LQAS as the government and partners in the country are scaling up its use.

The novelty of the current proposal is that it represents a combination of several tools previously implemented individually (often at national level) in an integral way in order to systematically address bottlenecks on supply and demand sides to implement strategies to reduce child morbidity and mortality at district level. It differs from previous approaches in that it is focused on priorities, on specific bottlenecks to implementation, and on local managerial gaps and evidence-based solutions. The lessons learned on strengthening district-level managerial capacities in both existing and new districts as well as on mechanisms for community monitoring will have implications not only in Uganda but also in other similar settings. To motivate early adoptions of lessons learned, CODES has a Steering Committee chaired by the Ministry of Health and sits quarterly to share experiences.

### Trial status

By the time of submitting this manuscript, the trial was at the intervention stage having successfully implemented the proof-of-concept stage during the first 2 years. Currently the intervention is in its final year.

## Abbreviations

ANCOVA: analysis of covariance
BNA: Bottleneck Analysis
CBOs: Community-based Organizations
CFI: Child Fund International
CODES: Community and District Empowerment for Scale-up
CQI: Continuous Quality Improvement
DBA: District Bottleneck Analysis
DHMT: District Health Management Team
FGD: focus group discussions
GEE: generalized estimating equations
LiST: Lives Saved Tool
LQAS: Lot Quality Assurance Survey
LSTM: Liverpool School of Tropical Medicine
MDGs: Millennium Development Goals
MFI: Model For Improvement
PPS: Probability Proportionate to Size
SSA: sub-Saharan Africa
UBoS: Uganda Bureau of Standards
UNICEF: United Nations Children’s Emergency Fund
WHO: World Health Organization
